# Evaluation of treatment costs for direct versus stepwise admission to home treatment

**DOI:** 10.3389/fpsyt.2025.1623610

**Published:** 2025-09-08

**Authors:** Antonia Kaehlitz, Michel Ngoc-Minh Dang, Jens Kronschnabel, Eva-Maria Pichler, Urs Hepp, Marc Walter, Maxim Zavorotnyy

**Affiliations:** ^1^ Psychiatric Services Aargau, Academic Hospital of Zurich University, Windisch, Switzerland; ^2^ Department of Consultation-Liaison Psychiatry and Psychosomatic Medicine, University Hospital Zurich, University of Zurich, Zurich, Switzerland; ^3^ hepp-health GmbH, Zurich, Switzerland; ^4^ Department of Psychiatry and Psychotherapy, Basel University, Basel, Switzerland; ^5^ Department of Psychiatry and Psychotherapy, Marburg University, Marburg, Germany

**Keywords:** home treatment, cost-effectiveness, psychiatric services, inpatient care, health economics

## Abstract

**Background:**

Mental health care’s rising socio-economic relevance has led to a need for cost-effective treatment alternatives. Home Treatment (HT) has emerged as a viable substitute for inpatient psychiatric care, introduced by the Psychiatric Services Aarau AG (PDAG) in 2015. Subsequent studies have evaluated its impact on hospital bed usage and treatment costs. This study aimed to assess the long-term effects of HT after its full integration into routine psychiatric care.

**Methods:**

An observational study included patients who received HT between 2019 and 2020. They were followed for two years, comparing cumulative costs, treatment duration, and readmission rates with a matched inpatient control group. Subgroup analyses distinguished between patients directly admitted to HT and those transitioning from inpatient care. Statistical analyses included Wilcoxon signed-rank tests and Fisher’s exact tests.

**Results:**

HT patients had fewer inpatient days but longer total treatment durations; costs did not differ significantly. However, analyses revealed that direct admission to HT was linked to a 24% cost reduction and a lower readmission rate compared to inpatient care. In contrast, combining inpatient care with HT led to increased treatment durations and costs.

**Conclusion:**

HT as a standalone treatment showed cost efficiency and reduced readmission rates, positioning it as a promising alternative to inpatient care. However, combining HT with inpatient treatment increased duration and costs, undermining the financial benefits. Future research should identify patient groups that benefit most from direct HT admission and explore hybrid models integrating short-term inpatient interventions followed by HT to enhance cost-effectiveness and clinical outcomes.

## Introduction

In recent years, mental health has become increasingly significant from both medical and socio-economic perspectives (1–3). As health care systems worldwide face growing financial pressures, newly introduced treatment approaches must be both effective and cost-efficient. This is highly relevant for the Swiss healthcare system, where according to recent estimates, 14.3% of costs are due to mental illnesses (4). Home Treatment (HT) has been discussed as an alternative to inpatient treatment for decades (5–7). It has now been implemented in various health care systems throughout the globe (8–10). Multiprofessional teams visit the patient at home on up to six days per week and offer a 24-hour availability for emergencies. These teams consist of doctor, psychologist, social workers, nurses and occupational therapists. This form of treatment is intended to be equivalent to inpatient treatment while offering certain advantages, such as reduced stigma towards psychiatric care, better involvement of the patient’s social environment, and accessibility for specific patient groups like caregivers of children.

In 2015, HT was introduced at the Psychiatric Services Aargau AG (PDAG) accompanied by an initial 12 month long randomized controlled trial (RCT) (11), examining to what extent the HT service would reduce hospital bed use. Patients were randomized into the service models including optional HT or to a conventional service model with inpatient treatment only. With HT involvement, the study showed a reduction of inpatient treatment within 24 months after the index crisis necessitating hospital admission in the HT group by over 30% with similar treatment outcome measured among others by the Health of the Nation Outcome Scale (12). Treatment costs in the HT group remained lower, however, not significantly, compared to the control group.

A follow-up study (13) examined whether patient characteristics and outcomes changed after HT was implemented into routine care, allowing patients to access this form of treatment without unnecessary barriers such as randomization. While a reduction in hospital bed use was still observed, it was less pronounced than during the RCT, and overall treatment duration increased. Both studies also compared demographic and clinical profiles between HT and inpatient groups, providing insights into which patients might benefit most from HT. Compared to patients treated within the RCT framework, those receiving home treatment under routine care more often lived with others, had less often been admitted compulsorily to acute care and stress-related and anxiety disorders were more prevalent. There have been other studies on the implementation of HT (14, 15) at other Swiss hospitals.

In the first two years of HT (2015 – 2017) at the PDAG, patients were admitted to HT either after walk-in at our emergency department or being referred to HT exclusively from inpatient treatment within the PDAG. Since 2017, referrals to HT can be initiated directly by outpatient practitioners or an inpatient ward of PDAG. Thus, we suppose that the diversification of admission pathways may influence treatment trajectories, associated costs, and patient characteristics. The aim of this study was to evaluate whether HT, after being integrated into routine psychiatric care at PDAG for several years, continues to reduce inpatient treatment costs in real-life conditions. We hypothesized that inpatient treatment durations will decrease in all HT groups compared to their respective inpatient control groups. Additionally, we anticipate that different treatment pathways might affect the extent of cost reduction. Based on previous findings (11), we expect that HT combined with inpatient treatment will not yield significant cost advantages due to increased cumulative treatment durations. Finally, we expect that direct admission to HT will reduce overall treatment costs and result in shorter treatment durations compared to standard inpatient care.

## Methods

The study was conducted at the Psychiatric Services Aargau AG (PDAG), one of the largest psychiatric clinics in Switzerland and the only facility in the Canton of Aargau providing mandated emergency psychiatric services (16). The study was designed as an observational study, including patients who received home treatment (HT) in the years 2019 or 2020. Patients were followed for two years, with the study concluding at the end of 2022. During this time, the number of inpatient and HT episodes and the associated costs were analyzed. This data was compared with a matched control group, based on age, gender, primary diagnosis, and the number of psychiatric comorbidities, which received solely inpatient treatment.

Patients’ sociodemographic, clinical data and service use were drawn from electronic medical records and from the case register using the hospital information system ORBIS NICE (Version: DACHL_08042700; Dedalus Healthcare group), which is routinely used in the PDAG. Data of all patients being admitted between January 1^st^, 2019, and December 31^st^, 2020, were analyzed over a follow-up period of two years. The first admission between January 1^st^, 2019, and December 31^st^, 2020, served as the starting point for the two-year follow-up, and provided sociodemographic data as well as the principal diagnosis. Patients with dementia, delirium, and substance use disorder (as a principal diagnosis) were excluded from HT and the study. Participants’ ages ranged from 18 to 66 years, and we also excluded those receiving services covered only by Swiss private or supplementary insurance. In line with the clinical care structures at PDAG, inpatient care during the study period was provided in psychiatric acute care units and in diagnosis-specific wards, ensuring differentiated treatment pathways for various patients’ needs.

The outcome measures were the treatment costs, the total amount of treatment days, and number of readmissions during the two-year period. In line with Swiss DRG (Diagnosis-Related Groups) guidelines (17) and in accordance with Swiss hospital billing regulations, a readmission within the first 18 days after release was not counted as a readmission but as the same stay. The overall costs were calculated by multiplying the number of inpatient treatment days by the daily DRG base rate (CHF 695, approximately EUR 720) or by multiplying the number of HT days by the HT rate, which was negotiated between health insurers, the canton, and PDAG (CHF 410, approximately EUR 425). Finally, the costs of inpatient and home treatment over the two-year period were added up.

The regional ethics committee determined that this research project does not fall under the Human Research Act, as it only evaluated anonymized clinical routine data. However, the committee confirmed that the project meets general ethical and scientific standards for human research.

### Definition of groups and subgroups

A total of 392 patients were included in the study. The HT all group was divided into two subgroups: HT - 1 and HT - 2, each with corresponding control groups. The HT all group encompasses all cases receiving HT during the observation period, excluding those who met the exclusion criteria. The HT - 1 subgroup consisted of patients directly admitted to HT after a preliminary consultation, with no inpatient stay in the 31 days prior to admission. This group included 90 patients, matched with an equally sized control group. The HT - 2 subgroup comprised of patients who received inpatient treatment before transitioning to HT, having had an inpatient stay 31 days prior to starting HT. This group included 106 patients, also matched with an equivalently sized control group. Allocation to the HT - 1 or HT - 2 subgroup was based solely on initial treatment within the two-year period. Subsequent inpatient or home treatments in both subgroups were classified as readmissions.

### Matching procedure for control group and subgroups

For both subgroups (HT - 1 and HT - 2) a control group was extracted from the clinical database by means of matching controls. Matched controls did not have any HT during a two-year period. These patients received inpatient treatment as usual. Matching criteria were gender, age (with a tolerance of +/- 5 years), the principal diagnosis (up to the 5^th^ character of ICD-Codes, i.e., Fxx.x), as well as the number of secondary diagnoses (with a tolerance of +/- 1 diagnosis). For around 11% of the HT patients, no pair with matching principal diagnoses up to the 5^th^ character could be found. In these cases, the length of the ICD code was reduced until a match was found (however, in all cases at least the first two characters matched ensuring the matched diagnoses belong to the same broad category of illness). The number of secondary diagnoses was taken from each patient’s stay with the largest number of recorded secondary diagnoses and served as an additional (besides ICD code) estimate for case complexity. The matching was based on the HT cases and from these the control group was formed.

### Data analysis

First, all HT cases were compared to the matched control group. In a second step, the two subgroups (HT - 1 and HT - 2) were compared to their respective matched control groups. The primary outcome measure was treatment costs, which were determined by the treatment duration and form of treatment. The secondary measure was the readmission rate within the 2-year-follow-up period. Since none of the outcome measures were normally distributed, Wilcoxon signed-rank tests were used to compare continuous variables of the matched groups. Discrete variables were compared using Fisher’s exact tests. *P*-values for all outcome measures were adjusted for multiple comparisons by means of the Benjamini-Hochberg method (18). Statistical significance was set at α = .05 and *p*-values were one-tailed in case of directed hypotheses (two-tailed otherwise). Statistical analyses were performed using R statistical software, version 4.2.1 (19).

## Results

Among the 392 patients in the study, 306 (78.1%) were female, and 312 (79.6%) held Swiss citizenship. The median age was 39 years (IQR = 32 – 50, range = 18 – 66). The most common primary diagnosis were mood (64.8%) and anxiety disorders (19.9%). Most patients lived in shared households (67.3%), and 38.5% were married. **Table 1** presents the sociodemographic and clinical characteristics of the different study groups. Matched characteristics such as gender, age, primary diagnosis, and number of secondary diagnoses did not show significant group differences. **Table 2** provides a comparison of treatment costs and cost-relevant characteristics. Comparison between both HT subgroups is summarized in **Supplementary Table S1** (in supplements).

**Table 1:** Characteristics of home treatment and control groups.

Characteristics	All (n=392)			Subgroup HT - 1 (n=180)			Subgroup HT - 2 (n=212)		
	HT all	Control	*p*-value*	HT-1	Control	*p*-value*	HT-2	Control	*p*-value*
Female, n (%)	153 (78.1)	153 (78.1)	>.9	67 (74.4)	67 (74.4)	>.9	86 (81.1)	86 (81.1)	>.9
Median age, yr (IQR)	39.0 (32.0 - 50.0)	39.0 (32.8 - 50.0)	.791	39.0 (30.2 - 48.8)	39.0 (31.0 - 48.0)	.830	39.0 (33.0 - 50.0)	39.5 (34.0 - 50.8)	.632
Swiss nationality, n (%)	164 (83.7)	148 (75.5)	.060	80 (88.9)	70 (77.8)	.071	84 (79.2)	78 (73.6)	.419
Civil status, n (%)			.158			.698			.118
single	76 (38.8)	82 (41.8)		39 (43.3)	40 (44.4)		37 (34.9)	42 (39.6)	
married	85 (43.4)	66 (33.7)		31 (34.4)	24 (26.7)		54 (50.9)	42 (39.6)	
married, but separated	8 (4.1)	16 (8.2)		5 (5.6)	5 (5.6)		3 (2.8)	11 (10.4)	
divorced	23 (11.7)	24 (12.2)		12 (13.3)	15 (16.7)		11 (10.4)	9 (8.5)	
widowed or unknown	4 (2.0)	8 (4.1)		3 (3.3)	6 (6.7)		1 (0.9)	2 (1.9)	
Residence prior to admission, n (%)			<.001			<.05			<.01
home, with others	149 (76.0)	115 (58.7)		67 (74.4)	54 (60.0)		82 (77.4)	61 (57.5)	
home, alone	40 (20.4)	51 (26.0)		22 (24.4)	25 (27.8)		18 (17.0)	26 (24.5)	
residential/retirement/care home		9 (4.6)			4 (4.4)			5 (4.7)	
other°		6 (3.1)			1 (1.1)			5 (4.7)	
unknown	7 (3.6)	15 (7.7)		1 (1.1)	6 (6.7)		6 (5.7)	9 (8.5)	
Primary ICD - 10 diagnosis, n (%)			>.9			>.9			>.9
F0	1 (0.5)	1 (0.5)					1 (0.9)	1 (0.9)	
F2	15 (7.7)	15 (7.7)		5 (5.6)	5 (5.6)		10 (9.4)	10 (9.4)	
F3	127 (64.8)	127 (64.8)		62 (68.9)	62 (68.9)		65 (61.3)	65 (61.3)	
F4	39 (19.9)	39 (19.9)		17 (18.9)	17 (18.9)		22 (20.8)	22 (20.8)	
F5	5 (2.6)	5 (2.6)		2 (2.2)	2 (2.2)		3 (2.8)	3 (2.8)	
F6	9 (4.6)	9 (4.6)		4 (4.4)	4 (4.4)		5 (4.7)	5 (4.7)	
Median number of secondary diagnoses (IQR)	1.0 (0.0 - 2.0)	1.0 (0.0 - 2.0)	.829	1.0 (0.0 - 2.0)	1.0 (0.0 - 2.0)	>.9	1.0 (0.0 - 2.0)	1.0 (0.0 - 2.0)	>.9
Involuntary admission, n (%)	12 (6.1)	29 (14.8)	<.01	2 (2.2)	11 (12.2)	<.05	10 (9.4)	18 (17.0)	.155

F0: Mental disorders due to known physiological conditions.

F2: Schizophrenia, schizotypal, delusional, and other non-mood psychotic disorders.

F3: Mood [affective] disorders.

F4: Anxiety, dissociative, stress-related, somatoform and other nonpsychotic mental disorders.

F5: Behavioral syndromes associated with physiological disturbances and physical factors.

F6: Disorders of adult personality and behavior.

HT, Home Treatment.

IQR, interquartile range.

*Wilcoxon signed-rank test for continuous data; Fisher’s exact test for categorical data; all values two-tailed.

°other includes, e.g., homeless or prison.

**Table 2:** Results of Wilcoxon signed-rank tests for HT groups compared to controls.

Characteristics	HT: Median (IQR)	Control: Median (IQR)	*n*	*W*	*Z*-score	*P*-value*	Effect size *r*
HT all							
Median all treatment days	46.0 (31.0 - 67.0)	30.0 (10.0 - 71.8)	392	11770	2.95	<.005 (1-tail)	.21
Median inpatient days only	7.5 (0.0 - 29.0)	30.0 (10.0 - 71.8)	392	4608	-6.27	<.001 (1-tail)	.45
Median treatment costs, in CHF (000s)	20.3 (13.5 - 33.7)	20.8 (7.0 - 49.9)	392	9348	-0.38	.702 (2-tail)	.03
Median number readmissions	0.0 (0.0 - 1.0)	0.0 (0.0 - 1.0)	392	2184	-0.86	.465 (2-tail)	.09
Subgroup HT - 1							
Median all treatment days	38.0 (28.0 - 50.0)	29.5 (10.0 - 68.8)	180	1994	-0.22	.465 (1-tail)	.02
Median inpatient days only	0.0 (0.0 - 0.0)	29.5 (10.0 - 68.8)	180	282	-7.11	<.001 (1-tail)	.75
Median treatment costs, in CHF (000s)	15.6 (11.5 - 21.2)	20.5 (7.0 - 47.8)	180	1323	-2.92	<.005 (1-tail)	.31
Median number readmissions	0.0 (0.0 - 0.0)	0.0 (0.0 - 1.0)	180	222	-2.34	<.05 (2-tail)	.31
Subgroup HT - 2							
Median all treatment days	56.0 (37.0 - 94.2)	30.0 (10.0 - 73.2)	212	3998	4.11	<.001 (1-tail)	.40
Median inpatient days only	19.5 (10.0 - 60.8)	30.0 (10.0 - 73.2)	212	2286	-1.59	.084 (1-tail)	.15
Median treatment costs, in CHF (000s)	28.3 (17.9 - 55.0)	20.9 (7.0 - 50.9)	212	3509	2.12	.058 (2-tail)	.21
Median number readmissions	0.0 (0.0 - 1.0)	0.0 (0.0 - 1.0)	212	988	0.77	.465 (2-tail)	.07

CHF: Swiss Frank.

HT, Home Treatment.

IQR, interquartile range.

*p-values are stated one- or two-tailed as indicated and all are adjusted for multiple comparisons (Benjamini-Hochberg procedure).

### HT all versus controls

**Figure 1:**
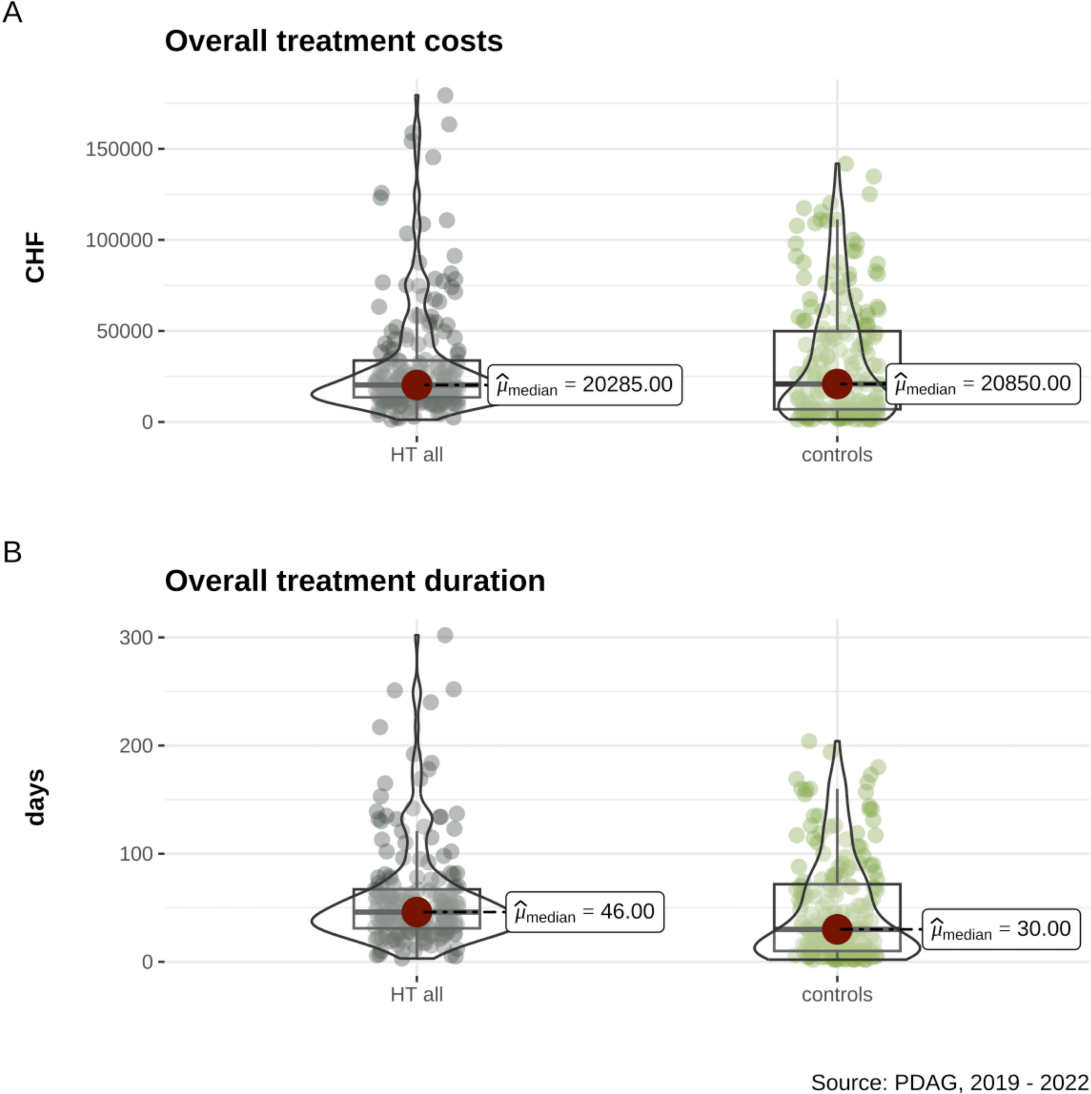
Comparisons between the Home Treatment (HT) group and controls matched based on age, gender, first diagnoses, and number of comorbid psychiatric diagnoses include: **(A)** overall costs in Swiss Francs (CHF) and **(B)** overall treatment duration in days.

Comprehensive statistical details are provided in **Tables 1** and **2** and depicted in **Figure 1**. As hypothesized, the cumulative treatment duration was significantly longer in the HT all group compared to the control group (**Figure 1B**). As expected, the HT all group had fewer inpatient treatment days than the controls. However, costs did not differ significantly between the two groups (**Figure 1A**), and the number of readmissions showed no significant difference. Notable differences in sociodemographic characteristics were observed between the groups (**Table 1**). Fisher’s exact test for residence prior to admission indicated that a greater proportion of patients in the HT all group lived in shared households compared to the controls. Additionally, the proportion of involuntary admissions was significantly higher in the control group.

### HT-1 versus controls

**Figure 2:**
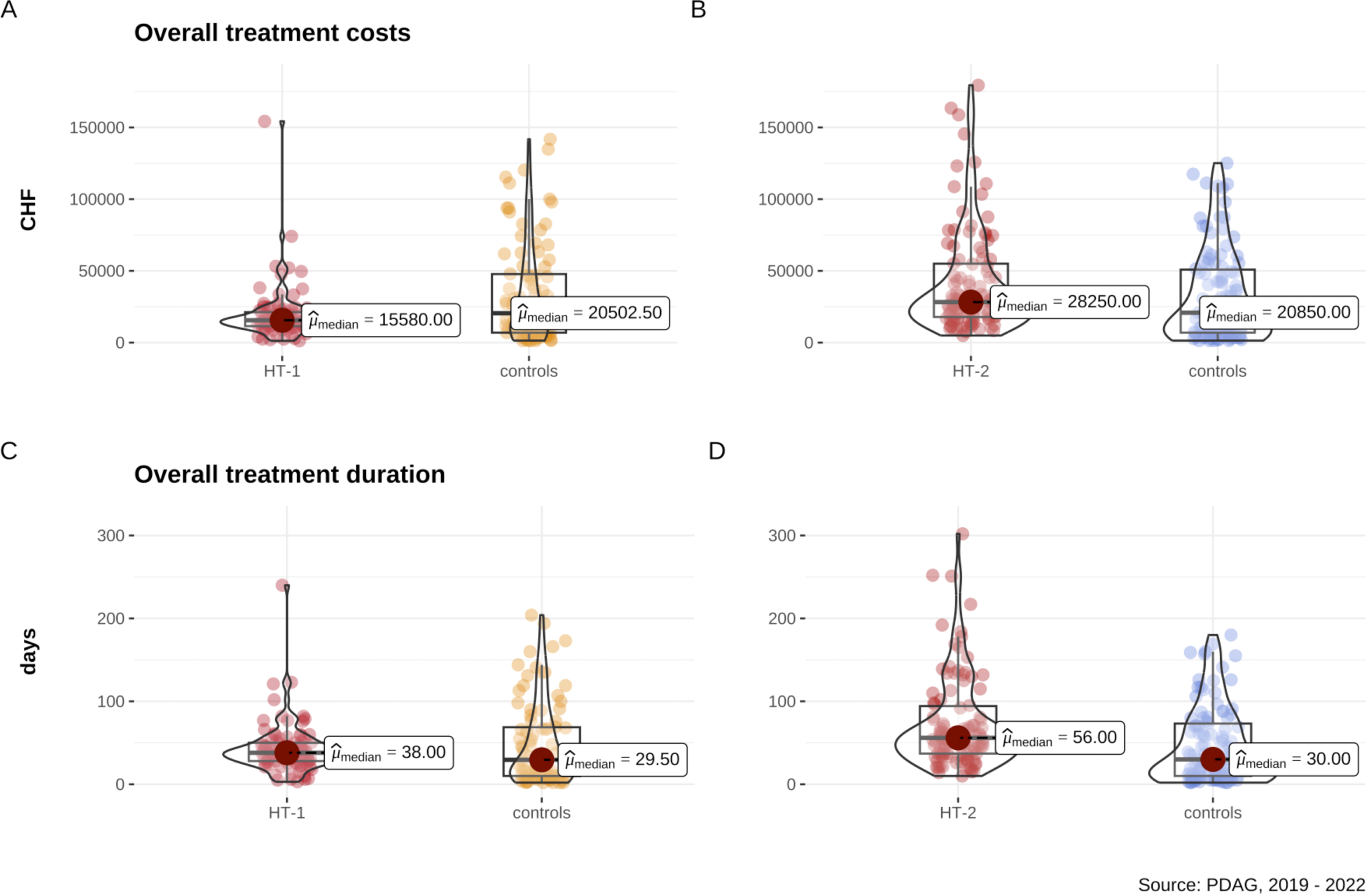
Analysis of subgroups reveals that overall costs in Swiss Francs (CHF) are **(A)** non-significantly lower in the subgroup of patients directly admitted to Home Treatment (HT - 1) and **(B)** significantly higher in the subgroup including patients who was treated first inpatient and then combined with Home Treatment (HT - 2) compared to matched controls based on age, gender, first diagnoses, and number of comorbid psychiatric diagnoses. **(C)** Overall treatment duration in days is non-significantly different in the HT - 1 subgroup and **(D)** significantly longer in the HT - 2 subgroup.

At the time of previous publications, direct admissions to home treatment were possible for only a small percentage of the study sample, making analyses of this subgroup particularly important for the current study. Our results suggest that treatment costs were significantly lower in the HT - 1 group compared to controls (**Figure 2A**), with HT - 1 treatment being 24% cheaper than inpatient treatment (effect size r = .31, indicating a medium effect). While the sum of treatment days was descriptively higher in HT - 1 than in controls, this difference was not statistically significant (**Figure 2C**). The mean sum of treatment days for HT - 1 was 36.99, including 6.12 inpatient days, reflecting a significant reduction of 87.54% in inpatient bed usage compared to controls. The readmission rate was also significantly lower for HT - 1, with a medium effect size (r = .31). Details are presented in **Table 2**. As shown in **Table 1**, groups differed in residence prior to admission, with more HT - 1 patients living in shared households compared to controls. Additionally, the percentage of patients experiencing involuntary admissions was markedly lower in HT - 1 than in the matched control subgroup.

### HT-2 versus controls

Patients in the HT - 2 subgroup were initially admitted to inpatient treatment, followed by a transfer to HT within 31 days after discharge. As shown in **Table 2** and **Figure 2B**, treatment costs for the HT - 2 group were 26.47% higher than in the control subgroup, although this difference did not achieve statistical significance after correction for multiple comparisons. The cumulative treatment duration in the HT - 2 subgroup was significantly longer than in the control subgroup, representing a strong effect (r = .40). Contrary to expectations, there was only a statistical trend toward reduced inpatient treatment days for HT - 2 compared to controls. Readmission rates did not differ significantly between the subgroups. In terms of sociodemographic characteristics, a higher percentage of HT - 2 patients lived in shared households compared to controls, with no significant differences in involuntary admissions.

## Discussion

Consistent with previous studies (20, 21), our findings indicate that, when implemented in routine clinical practice, home treatment (HT) does not result in a significant cost advantage over inpatient care. Specifically, HT patients experienced fewer inpatient days but had longer total treatment durations, which offset potential cost savings. Overall, costs did not differ significantly between groups. A similar pattern has been reported in studies on psychiatric day hospital treatment, another alternative to inpatient care, where greater symptom improvement and treatment satisfaction were observed, but treatment durations and associated costs were generally higher in the day hospital group (22).

This prompted us to further explore whether different trajectories of HT application have distinct effects on treatment costs. To this end, we conducted a cost analysis across subgroups, differentiating between patients who received HT as a primary treatment (HT - 1) and those who transitioned to HT following an initial inpatient stay (HT - 2). The results suggest that combining inpatient care with subsequent HT, as in the HT - 2 subgroup, leads to significantly longer cumulative treatment duration and, consequently, higher overall treatment costs. These results are in line with previous reports from the subsequent follow-up study after HT was implemented in routine care (13), where an increase in total treatment days was observed during the second year, after the initial RCT (12) was completed and HT was implemented into routine care. Recent studies primarily examined how HT as a main treatment could reduce inpatient capacity usage (8, 20 – 22), potentially lowering costs. The results from the HT - 1 subgroup support these findings. In contrast, combining inpatient care with subsequent HT, as in the HT - 2 subgroup, resulted in significantly longer cumulative treatment duration and, consequently, higher overall treatment costs. This pattern diverges from the findings of Stulz et al., who, in the initial RCT, reported a 30% reduction in inpatient days, leading to a shorter total treatment duration. Our current results suggest that, although HT holds potential for reducing inpatient stays, this effect is challenging to sustain in routine clinical practice, particularly when HT follows an initial inpatient treatment. However, HT as the primary mode of care (HT - 1) resulted in an 87.5% reduction in inpatient bed utilization and a 24% median cost reduction compared to controls over the same two-year period. Contrary to our initial hypothesis, which suggested that the HT - 1 group would have a shorter overall treatment duration, the total treatment duration (sum of all inpatient and HT days during the two-year follow-up) was non-significantly longer than that of the solely inpatient-treated controls. However, it was accompanied by a significantly lower readmission rate, suggesting potential long-term stabilizing effects of home treatment when applied early and proactively.

Patient characteristics and contextual factors may also play a critical role in explaining the observed differences in treatment duration and costs. In particular, the proportion of involuntary admissions differed notably between the subgroups. Involuntary admission may indicate a severe level of illness due to acute risk to oneself or others. In this study, the HT - 1 subgroup had significantly fewer involuntary admissions than the HT - 2 subgroup. Along with a higher readmission rate in the HT - 2 subgroup, this suggests that HT - 1 patients may exhibit more favorable illness characteristics and therefore require shorter treatment durations. This interpretation aligns with Mötteli et al., who reported that lower symptom severity and current employment status are associated with a higher likelihood of successfully substituting inpatient treatment with HT (23). Additionally, legal formalities associated with involuntary admissions may potentially prolong inpatient treatment in certain cases. However, Swiss and cantonal regulations mandate that the criteria for involuntary treatment be reviewed daily. If these criteria are no longer met, patients should be discharged upon request, which is likely to reduce the potential prolongation mentioned earlier. Overall, these results support the need for subgroup differentiation, reinforcing that HT - 1 patients exhibit distinct characteristics compared to those in the HT - 2 group.

While patient characteristics and legal factors are important in understanding treatment duration and costs, from a clinical perspective, the transition from inpatient to outpatient treatment presents additional challenges and considerations. Sometimes, it can lead to a renewed worsening of symptoms and a possible readmission after initial discharge (24, 25). For these cases HT enables an intensive form of treatment, at the same time allowing patients to become reacquainted with their home setting. Our data show that this clinical practice does not necessarily offer significant cost advantages. But the involvement of HT could mean that the patient can be discharged earlier, thereby freeing up capacities in inpatient units. However, it must be considered that each individual case needs to be assessed to determine whether such an intensive form of post inpatient treatment is necessary or if other, less intensive outpatient treatment options would suffice. If feasible, a transfer to HT should be considered shortly after hospital admission.

Building on the clinical considerations discussed earlier, operational factors during the transition from inpatient care to home treatment, such as the change in treatment team, may also influence treatment duration and costs. Establishing a new therapeutic relationship requires time and may delay treatment progress. In this context, ensuring therapeutic continuity is not only essential for clinical outcomes but could also have economic implications. Repeated patient admissions and disruptions in care processes tend to prolong treatment episodes and increase overall costs, particularly as the initial days of hospitalization are associated with the highest per diem costs due to more intensive ancillary services (26). Therefore, an integrative approach, where the patient is continuously treated by the same team (both inpatient and HT), might potentially reduce treatment duration and thus lower the costs. This emphasis on team continuity aligns with the Open Dialogue approach, which promotes consistent therapeutic involvement across all phases of care. By maintaining a stable treatment team, Open Dialogue addresses fragmented care pathways and supports more efficient treatment processes in psychiatric care settings (27).

This study also confirmed the HT-patient characteristics previously described in other studies, one of them being mood disorders as the most common primary diagnosis (28). Secondly, while matching ensured, that both HT and control samples had an identical number of women, the HT sample characteristics indicate that women utilized HT more frequently (78% vs. 22% men), aligning with previous studies (23). This may be attributed to the frequent referrals of female patients with postpartum depression and psychosis, for which HT is well-suited. Anxiety disorders are more prevalent in women, with studies indicating that they are approximately twice as likely as men to experience these conditions (29). This higher prevalence may contribute to the increased referrals of anxiety disorders, as HT appears well suited for cognitive behavioral based interventions and for *in vivo* exposure embedded in the patients everyday environment (30).

As noted by Stulz et al. (13), a change in the characteristics of patients admitted to HT was observed after the transition from the randomized controlled trial phase in the first study year to non-randomized admissions in the second year. The authors of that study attributed this discrepancy to a potential Hawthorne effect (30), which describes the phenomenon where individuals alter their behavior due to the awareness of being observed or studied in an experiment, thereby impacting the outcomes. Since our study was conducted entirely under routine care conditions, without the structured procedures inherent to randomized trials, this difference appears to have persisted, particularly in the HT - 2 subgroup. While many studies have explored which patients benefit from HT (23, 28, 31, 32), future research could examine whether admission algorithms based on these criteria contribute to better outcomes and lower costs.

### Limitations

A primary methodological limitation of this study concerns the group matching process. A structured symptom severity rating, such as HoNOS, was only available for a small proportion of HT patients, preventing its inclusion as a matching criterion or as a measure of treatment success. Consequently, potential differences in clinical severity between groups could not be fully accounted for. To approximate clinical comparability, we matched groups based on the principal ICD - 10 diagnosis and the number of psychiatric comorbidities, with diagnoses extracted from the hospital information system and validated by a senior psychiatrist (33). For approximately 11% of patients, no exact match at the five-character ICD - 10 level was available, necessitating truncation until a comparable pair was found. To assess potential bias resulting from this reduced specificity, we repeated all primary analyses excluding these cases, which yielded identical patterns of statistical significance and interpretation. Furthermore, to address possible confounding due to illness severity, partially reflected in the rate of involuntary admissions, we conducted subgroup analyses excluding all involuntarily admitted patients. Both sensitivity analyses confirmed the robustness of the main findings, suggesting that despite these methodological limitations, the applied matching strategy provided sufficient clinical comparability between groups. We believe that including symptom severity ratings in the matching process would not have significantly affected our findings, though it would have provided additional insights into patient distributions in a real-world setting.

Another limitation is that, due to Swiss data protection laws, we could only gather data on treatments at PDAG, making it impossible to rule out care received at other hospitals during the study period. Also, HT services at the PDAG were not available for individuals over 66 so this subpopulation was excluded. Additionally, we could not include costs for outpatient treatments provided by external services. Since PDAG is the only hospital offering acute psychiatric treatment and Home Treatment (HT) in the Canton of Aargau, our analyses focused on acute psychiatric treatment, excluding costs from day clinics. Two years of the study period coincided with the COVID - 19 pandemic. However, both treatment forms (home treatment and inpatient care) continued during this time. As both were equally affected, we do not expect this to have systematically influenced the comparative outcomes. Moreover, only two of the four years of the observation period were impacted by the pandemic, further minimizing a potential bias.

## Conclusion

In accordance with previous studies, HT in the Swiss Canton of Aargau may effectively reduce treatment costs. Its efficacy, assessed in terms of treatment duration, cost efficiency, and readmission rates, is optimized when HT is utilized as a standalone intervention rather than in conjunction with inpatient treatment, which diminishes the inherent benefits of HT. From an economic viewpoint, we therefore suggest that HT be employed primarily as an inpatient-equivalent intervention, designated for cases where outpatient or day clinic care proves inadequate, thereby mitigating the risk of unintended expansion of service volumes. Subsequent studies should explore which patients or diagnostic groups may benefit from direct admission to HT without prior inpatient care, potentially enhancing its overall effectiveness. Furthermore, the implementation of a program that provides short-term inpatient crisis intervention followed by HT may yield significant benefits for both patients and healthcare systems.

## Data Availability

The raw data supporting the conclusions of this article will be made available by the authors, without undue reservation.
