# Supplementary material for: Evaluation of treatment costs for direct versus stepwise admission to home treatment
Source: Front Psychiatry. 2025 Sep 8;16:1623610. doi: 10.3389/fpsyt.2025.1623610 (PMC12450985; doi:10.3389/fpsyt.2025.1623610)
Supplement: Supplementary file 1 [file Table1.docx]

**Table S1 Comparison of characteristics and treatment data of home treatment groups (without controls)**

|  |  | **HT all**  **(n=196)** | **HT-1**  **(n=90)** | **HT-2**  **(n=106)** | ***p*-value* HT-1 vs. HT-2** |
| --- | --- | --- | --- | --- | --- |
| Female, n (%) | | 153 (78.1) | 67 (74.4) | 86 (81.1) | .300 |
| Median age, yr (IQR) | | 39.0 (32.0 - 50.0) | 39.0 (30.2 - 48.8) | 39.0 (33.0 - 50.0) | .283 |
| Swiss nationality, n (%) | | 164 (83.7) | 80 (88.9) | 84 (79.2) | .082 |
| Civil status, n (%) | |  |  |  | .150 |
|  | single | 76 (38.8) | 39 (43.3) | 37 (34.9) |  |
|  | married | 85 (43.4) | 31 (34.4) | 54 (50.9) |  |
|  | married, but separated | 8 (4.1) | 5 (5.6) | 3 (2.8) |  |
|  | divorced | 23 (11.7) | 12 (13.3) | 11 (10.4) |  |
|  | widowed or unknown | 4 (2.0) | 3 (3.3) | 1 (0.9) |  |
| Residence prior to admission, n (%) | |  |  |  | .122 |
|  | home, with others | 149 (76.0) | 67 (74.4) | 82 (77.4) |  |
|  | home, alone | 40 (20.4) | 22 (24.4) | 18 (17.0) |  |
|  | unknown | 7 (3.6) | 1 (1.1) | 6 (5.7) |  |
| Primary ICD-10 diagnosis, n (%) | |  |  |  | .858 |
|  | F0 | 1 (0.5) |  | 1 (0.9) |  |
|  | F2 | 15 (7.7) | 5 (5.6) | 10 (9.4) |  |
|  | F3 | 127 (64.8) | 62 (68.9) | 65 (61.3) |  |
|  | F4 | 39 (19.9) | 17 (18.9) | 22 (20.8) |  |
|  | F5 | 5 (2.6) | 2 (2.2) | 3 (2.8) |  |
|  | F6 | 9 (4.6) | 4 (4.4) | 5 (4.7) |  |
| Median number of secondary diagnoses (IQR) | | 1.0 (0.0 - 2.0) | 1.0 (0.0 - 2.0) | 1.0 (0.0 - 2.0) | .678 |
| Compulsory admission, n (%) | | 12 (6.1) | 2 (2.2) | 10 (9.4) | <.05 |
| **Treatment data (IQR)** | |  |  |  |  |
|  | Median all treatment days | 46 (31.0 - 67.0) | 38 (28.0 - 50.0) | 56 (37.0 - 94.2) | <.001 |
|  | Median inpatient days only | 7.5 (0.0 - 29.0) | 0 (0.0 - 0.0) | 19.5 (10.0 - 60.8) | <.001 |
|  | Median treatment costs, in CHF (000s) | 20.3 (13.5 - 33.7) | 15.6 (11.5 - 21.2) | 28.3 (17.9 - 55.0) | <.001 |
|  | Median number readmissions | 0.0 (0.0 - 1.0) | 0.0 (0.0 - 0.0) | 0.0 (0.0 - 1.0) | <.001 |

CHF: Swiss Frank

F0: Mental disorders due to known physiological conditions

F2: Schizophrenia, schizotypal, delusional, and other non-mood psychotic disorders

F3: Mood [affective] disorders

F4: Anxiety, dissociative, stress-related, somatoform and other nonpsychotic mental disorders

F5: Behavioral syndromes associated with physiological disturbances and physical factors

F6: Disorders of adult personality and behavior

HT: Home Treatment

IQR: interquartile range

*Wilcoxon rank-sum test for continuous data; Fisher’s exact test for categorical data; all values two-tailed.

°other includes, e.g., homeless or prison.
